# The Erythrocyte Fatty Acid Profile and Cognitive Function in Old Chinese Adults

**DOI:** 10.3390/nu8070385

**Published:** 2016-06-23

**Authors:** Linhong Yuan, Jie Zhen, Weiwei Ma, Can Cai, Xiaochen Huang, Rong Xiao

**Affiliations:** School of Public Health, Capital Medical University, Beijing 100069, China; ylhmedu@126.com (L.Y.); xiaorong22@ccmu.edu.cn (J.Z.); weiweizibeike@163.com (W.M.); ccfdzz1023@163.com (C.C.); xiaochenhuang@126.com (X.H.)

**Keywords:** erythrocyte fatty acids, lipids, cognition, the elderly

## Abstract

Objective: To explore the relationship between the erythrocyte fatty acid profile and cognition in elderly Chinese adults. Methods: 60 mild cognitive impairment (MCI) subjects and 60 age- and gender-matched control adults (aged 55 years and above) were involved in this cross-sectional study. Cognitive function was measured by using the Montreal Cognitive Assessment (MoCA) test. Information regarding the demographic characteristics and lifestyle of the participants was collected with a questionnaire. A semi-quantified food frequency questionnaire (FFQ) method was used for dietary assessment. The erythrocytes fatty acid profile was measured. Results: The MCI subjects had a lower education level than the control subjects (*p* < 0.05). Compared with control subjects, MCI subjects had higher daily poultry intake and lower fish intake (*p* < 0.05). Erythrocyte fatty acid profile of the MCI subjects was characterized as lower erythrocyte proportions of 20:4 *n*-6, 20:5 *n*-3, and total *n*-3 fatty acids compared with control subjects (*p* < 0.05). An association of erythrocyte proportions of 18:0, 22:0, total SFA, 18:2 *n*-6, 24:4 *n*-6 fatty acids, docosahexaenoic acid (DHA), and total *n*-6 PUFAs with cognition in elderly Chinese adults was detected. Conclusion: The erythrocyte fatty acid profile was related to cognitionin the elderly. Lower erythrocyte unsaturated fatty acid and higher saturated fatty acid proportions might predict cognitive function decline in elderly Chinese adults.

## 1. Introduction

Observational studies indicated that various food-derived nutrients might delay the onset of cognitive decline and dementia in the elderly. Recently, the possible role of fatty acids in preventing age-related cognitive decline and cognitive impairment caused extensive attention. An increasing body of epidemiological evidence suggests that elevated saturated fatty acids (SFA) could have negative effects on age-related cognitive decline and mild cognitive impairment (MCI) [1,2,3]. Furthermore, a clear reduction of risk for cognitive decline has been found in population samples with high intakes of polyunsaturated fatty acids (PUFAs) [4]. PUFAs are commonly named according to the position of double bonds and the total chain length. The term *n*-3 PUFAs indicates that, counting from the methyl (CH3) end of the molecule, the first double bond is located between the third and fourth carbons. For *n*-6 PUFAs, the first double bond is located between the sixth and seventh carbons. *n*-3 and *n*-6 PUFAs, as the predominant long-chain polyunsaturated fatty acids (LCPUFAs) in the mammalian brain and neural tissues, play important roles in maintaining normal brain functions in the elderly [5]. It is believed that *n*-3 PUFAs enable fluidity in neuronal membranes and help regulate neurotransmitters [6], both crucial for optimal brain function. Sufficient dietary intake of *n*-3 long-chain polyunsaturated fatty acids (LCPUFAs) has been proven to be correlated with a low risk of dementia. Plasma studies have provided evidence that low *n*-3 levels are associated with dementia. Eicosapentaenoic acid (EPA) and docosehexaenoie acid (DHA), the predominant *n*-3 LCPUFA of membrane phospholipids in mammalian brain and neural tissues, play key roles in maintaining optimal brain functions in the elderly. Conquer found lower levels of plasma phospholipid DHA in patients with Alzheimer’s disease and other dementias [7]. Sinn and co-workers’ double-blind, randomized controlled trial indicated that increased intakes of DHA and EPA benefited mental health in elderly people with MCI. Increasing *n*-3 PUFA intakes may reduce depressive symptoms and the risk of progression to dementia [8]. Chiu also found that the *n*-3 fatty acid supplements significantly improved the cognitive portion of the Alzheimer’s disease Assessment Scale (ADAS-cog) in MCI participants. The author also observed higher proportions of EPA on red blood cell (RBC) membranes, which were also associated with better cognitive outcome [9]. All these results suggest that the retention of adequate tissue levels of both *n*-3 LCPUFAs was essential to keep normal brain functions. For the prevention of age-related neuron degenerative diseases and the maintenance of brain function, the retention of adequate tissue levels of both *n*-3 PUFAs might be necessary.

It was reported that erythrocyte fatty acid levels were correlated with resulting fatty acids in the brain. Moreover, LCPUFA compositions in erythrocyte membranes and plasma are higher than those of other lipid fractions [6]. Previous studies indicated that the correlation between the LCPUFA level in brain tissue and blood was stronger in erythrocytes than in plasma [10,11]. The measurement of erythrocyte fatty acid levels might indirectly reflect the fatty acid status in brain tissues. Therefore, in order to explore the relationship between the erythrocyte fatty acid profile and cognition in the elderly, in the present study, we measured the erythrocyte fatty acid profile of MCI and cognition intact control subjects to compare the differences in the in vivo fatty acid profile. At the same time, a FFQ method was applied in the current study to collect the information of dietary fat-rich food intake of the MCI and control subjects with the objective of uncovering the association between dietary fat-rich food intake and the in vivo fatty acid profile in the elderly. Our study provides new insights that guide future studies and therapeutic approaches to cognition decline in elderly Chinese adults.

## 2. Method

### 2.1. Subjects

Sixty MCI subjects and 60 age- and gender-matched control subjects (aged 55 years and above) were recruited from our previous cross-sectional study carried out in Nanyuan Community, Fengtai District, Beijing, China. The criteria included no use of drugs that may affect lipid metabolism and no history of chronic diseases, such as ischemic heart disease, diabetes, liver diseases, kidney disease, malignant tumors, cerebral stroke, or a recent history of alcohol abuse or antioxidant supplementary. Subjects who used supplements including a large amount of fatty acid were excluded. Subjects with AD or Parkinson’s disease (PD), those who were unable to complete the cognition test, and those with a long-term intake of anti-depressants or medications acting on the central nervous system were also excluded from the study. Written informed consent was obtained from all participants. The study protocol was approved by the Human Ethics Committee of the Capital Medical University (No. 2012SY23). The procedures followed the ethical standards of the Helsinki Declaration of 1975. 

### 2.2. Cognitive Function Measurement

Medical doctors from the community health service center interviewed the participants face to face, and cognitive function was measured by using the Montreal Cognitive Assessment (MoCA), which assessed several cognitive domains including short-term memory recall ability, visual-spatial abilities, executive functions, phonemic fluency ability, verbal abstraction ability, attention, concentration and working memory, language, and orientation. This assessment was validated in the detection of mild cognitive impairment and early AD, and widely used in other large-scale studies on cognitive function in the elderly previously. According to a previous study conducted in an elderly Chinese population [12], the cut-off points used for MCI diagnosis were as follows: 13/14 for individuals with no formal education, 19/20 for individuals with 1 to 6 years of education, and 24/25 for individuals with 7 or more years of education. The cut-offs above were shown to be sensitive and efficient in the diagnosis of MCI in an elderly Chinese population. 

### 2.3. Dietary Assessment

Participants were visited at a community health service center by specifically trained nutritionists and nurses. A validated self-administered semi-quantitative food frequency questionnaire (FFQ) was used to assess the habitual consumption of 13 food groups (fruit and vegetable, whole grain, legume and legume product, red meat, light meat, fish, eggs, nuts, cooking oil, milk, coffee, fruit and vegetable juice, and tea, comprising 41 items in total). This questionnaire was adapted from the questionnaire used for the Dietary Investigation of Chinese Residents, which was organized by the Chinese Nutrition Society (CNS) in 2010 [13]. The food intake survey collected the information, including the consumption frequencies (daily and weekly) and the quantity of foods consumed.

### 2.4. Blood Sampling

Fasting venous blood samples was collected between 8:00 a.m. and 9:00 a.m. from each subject. A vacuum heparinized tube was used for the blood sampling. The samples were centrifuged at 1600 *g* for 10 min to separate plasma and erythrocytes. The plasma and erythrocytes obtained were used for the measurement of fatty acid composition in the membrane.

### 2.5. Plasma and Erythrocyte Parameters Measurement

An ILAB600 clinical chemistry analyzer (Instrumentation Laboratory) was used to determine plasma glucose (Glu), triglyceride (TG), and total cholesterol (TC). High density lipoprotein cholesterol (HDL-C) was measured by using commercially available assays from Instrumentation Laboratory (Massachusetts, MA, USA). Low density lipoprotein cholesterol (LDL-C) was calculated using the Friedewald formula [14]. All samples for each participant were analyzed within a single batch, and the inter-assay coefficients of variation (CV) were less than 5%.

### 2.6. Fatty Acid Analysis

Erythrocyte total lipids were extracted according to the method descriptor by Folch et al. [15]. To determine the proportions of fatty acids in the erythrocyte membrane, lipids were extracted from the stored RBCs. Erythrocyte membrane extraction was carried out by using vacuum tubes containing ethylene diamine tetra-acetic acid, according to the description of Dai et al. [16]. Fatty acid methyl esters from the erythrocyte membrane were obtained as described previously [17]. Individual fatty acids were identified by the comparison of retention time with known standards (Sigma-Aldrich Inc., St. Louis, MO, USA) and expressed as a percentage of the total fatty acids quantified from peak areas. The intra- and inter-assay CVs for all measurements were 5%.

### 2.7. Statistical Analyses

Data was analyzed with the software SPSS 19.0 (Chicago, IL, USA). All continuous variables are presented as means ± SD. Age, gender, living status, education level, smoking, physical activity, and cognitive activity were presented as category variables. A Student’s *t*-test was used to compare the means of the detected parameters between the groups. Correlation tests were performed according to Spearman’s rank. *p* < 0.05 was considered to be statistically significant.

## 3. Results

### 3.1. Demographic Characteristics

In total, 120 elderly Chinese adults (60 MCI subjects and 60 age- and gender-matched control subjects) were recruited in the present study. The demographic characteristics of the participants are listed in Table 1. The MCI subjects had a lower education level than the control subjects (*p* < 0.05); and most participants in control group had a primary or junior high school degree. The percentage of subjects who smoke or drink alcohol was not different between control and MCI subjects (*p* > 0.05). Moreover, we did not detect any differences in clinical parameters between control and MCI subjects (*p* > 0.05).

### 3.2. Lipid-Rich Food Intake

As shown in Table 2, the MCI subjects have a higher poultry intake than the cognitive function of intact control subjects; the differences in daily poultry intake between the groups reached statistical significance (*p* < 0.01). The control subjects consumed more fish daily than the MCI individuals (*p* < 0.05). Despite no statistical significance, the intake of red meat of MCI subjects was higher than that of the control subjects; and the control subjects had a higher legume intake than MCI subjects. 

### 3.3. Erythrocyte Fatty Acid Profile of MCI and Control Subjects

The erythrocyte fatty acid status of the participants is shown in Table 3. As compared with control subjects, the erythrocyte fatty acid profile of the MCI subjects was characterized as having a higher portion of 20:4 *n*-6 (arachidonic acid, AA), 20:5 *n*-3 (eicosapentaenoic, EPA) unsaturated fatty acids, and total *n*-3 PUFAs (*p* < 0.05). However, despite no statistical significance, we found that there was a reduction in 22:6 *n*-3 (docosahexaenoic acid, DHA) proportion, and an increase in the *n*-6/*n*-3 ratio of erythrocyte lipids in MCI subjects, compared with control individuals. 

### 3.4. Association between Erythrocyte Fatty Acids and Cognitive Function 

As shown in Table 4, the total SFA proportion among the total fatty acids of erythrocyte lipids was mainly inversely associated with the cognition of visual-spatial ability (*r* = −0.311, *p <* 0.01). Among the detected saturated fatty acids, the proportion of 12:0 fatty acid was mainly inversely associated with total MoCA score (*r* = −0.450, *p* *<* 0.05); the percentage of erythrocyte membrane 18:0 fatty acids was negatively related with delayed memory and orientation ability in the detected elderly (*r* = −0.334, *r* = −0.369, respectively, *p* *<* 0.05); the percentage of 22:0 fatty acid was inversely correlated with visual-spatial ability (*r* = −0.323, *p* *<* 0.05) and total MoCA score (*r* = −0.415, *p* *<* 0.05). The18:2 *n*-6 (linoleic acid, LA) unsaturated fatty acid was mainly positively associated with visual-spatial ability (*r* = 0.302, *p* *<* 0.05) and name ability (*r* = 0.305, *p* *<* 0.05). A strong positive correlation between the proportion of 24:4 *n*-6 fatty acid and total MoCA score was detected in the investigated elderly subjects (*r* = 0.434, *p* *<* 0.05). The percentage of total *n*-6 PUFAs were positively associated with visual-spatial ability (*r* =0.341, *p* *<* 0.05). The percentage of erythrocyte DHA was positive correlated with total MoCA score (*r* = 0.356, *p* *<* 0.05).No association was found between EPA, total PUFAs, the *n*-6/*n*-3 ratio, or cognitive performance in the investigated MCI subjects.

## 4. Discussion

Fatty acid intake and the circulating lipid profile might affect the development of cognitive impairment by way of the influence of fatty acids on atherosclerosis and thrombosis [18,19]. In the present study, we compared the lipid-rich food intake and erythrocyte lipid profile between MCI and control subjects. Our data indicated the difference of lipid-rich food intake and the erythrocyte lipid profile between MCI and control subjects. Our findings suggest that the difference in lipid-rich diet intake and changes in erythrocyte lipid status might be involved in the cognition decline in elderly Chinese adults.

In the current study, we found that the MCI subjects consumed more red meat, poultry, egg, and milk than the control subjects, and the difference in daily poultry consumption between groups reached statistical significance (*p* < 0.05). These results are consistent with other studies about dietary pattern and cognition in the elderly [20,21]. It is well-known that red meat and poultry are the main food resources of saturated fatty acids, which are closely related to the risk of cardiovascular diseases in elderly adults. Moreover, we detected that the cognition intact control subjects consumed more fish than the MCI subjects (*p* < 0.05). Prospective studies reported that the incidence of dementia and cognitive decline have been shown to be inversely related to dietary fish intake [22,23]. A fish-rich diet has been suggestive of cognition protection and efficiency in preventing further cognition decline in MCI patients such that AD develops. In particular, large-scale longitudinal studies showed that consumption of fish more than twice per week was associated with a reduction in the risk of dementia by 28% and of AD by 41%, in comparison with those who ate fish less than once per month [24]. However, a recent report from Vanessa Danthiir’s study indicated that increased fish intake has no beneficial effect on baseline cognitive performance in cognitively normal elderly adults. Instead, the authors detected a small negative effect of fish intake on elderly-age cognitive function [25]. The inconsistency of the relationship between fish intake and cognition in the elderly might be attributed to several factors, such as the dietary investigated method, the type of fish consumed, and the longitudinal associations between past fish intake and cognition. As for legume or legume products, despite no statistical significance, the daily legume and legume product intake of control subjects was higher than that of MCI patients. Together with other reports [26,27], our data indicates the possible beneficial effects of a legume- or legume-product-rich diet on cognitive function in the elderly. 

An increasing number of population-based studies have indicated an association of altered polyunsaturated fatty acid (PUFA) levels with cognitive decline [28]. Moreover, abnormal in vivo lipid status was reported to be associated with an enhanced possibility of dementia [29]. Therefore, in the present study, the erythrocyte lipid profiles of the MCI and control subjects were compared. Differences in *n*-6 and *n*-3 PUFA content in erythrocyte membrane total lipids were observed. As shown in Table 3, compared with MCI subjects, higher erythrocyte proportions of 20:4 *n*-6, 20:5 *n*-3, and total *n*-3 PUFAs were detected in control subjects. Except of EPA and DHA, our data also supported the contribution of other LCPUFAs to cognition in the elderly, which was demonstrated by a lower percentage of 24:4 *n*-6 PUFA in the total erythrocyte membrane lipids of MCI patients. These results were in agreement with a report by Cunnane and coworkers. In their study, the authors observed the differences in plasma fatty acid profiles in AD (or MCI) and control subjects were not specific to DHA or even to *n*-3 fatty acids [30]. Iuliano also reported that, compared with control subjects, the sum of plasma *n*-6 fatty acids was lower in AD than were the *n*-3 fatty acids [31]. These results consistently indicated that cognition decline in the elderly is associated with altered plasma or tissue status of both *n*-3 PUFAs and other fatty acids unrelated to DHA or EPA. Together with the dietary survey results from the present study, we speculate that the low intake of a LCPUFA-rich diet (such as fish) and abnormal fatty acid status, especially LCPUFAs, might altogether involve the occurrence of aging-associated cognition decline. 

Several studies have suggested that an increased intake of saturated fatty acids (SFA) could have negative effects on cognitive functions [32,33]. A series of longitudinal studies evaluated the possible relationship between dietary fatty acid intake and the risk of dementia and AD. In the Rotterdam study, the researchers suggested that an elevated intake of total lipids and saturated fat increased the risk of dementia with a cerebrovascular component [34]. In the current study, we found that the proportions of erythrocyte membrane 18:0 and 22:0, fatty acids and total SFA, were negative correlated with delayed memory, orientation, visual-spatial ability, and name ability, respectively in the elderly (*p* *<* 0.05). These data were consistent with Baym’s report about SFA intake and memory ability. The authors observed a negative association of SFA intake with relational (hippocampal-dependent) and item (hippocampal-independent) memory in children [35]. 

A high intake of *n*-6 polyunsaturated fatty acids, such as linoleic acid, can increase oxidative stress, and has been found to be positively associated with cognitive deficit [36]. Milte also reported a higher amount of erythrocytesin *n*-6 PUFAs in MCI patients than that in control subjects [28]. In contrast with these studies, in the present study, we detected an association between erythrocyte *n*-6 PUFAs (including 18:2 *n*-6, 24:4 *n*-6, and total *n*-6 PUFAs) and visual-spatial, name, orientation ability and total MoCA score in elderly subjects. This result was in line with González’s study, in which the author found that the *n*-6/*n*-3 polyunsaturated fatty acid ratio of the PUFA intake was positively related to the MMSE score in elderly adults aged 75 years and above [37]. In fact, in the Three City study, a higher plasma ratio of 20:4 *n*-6 (arachidonic acid, AA) to DHA was associated with an increased risk of incident dementia, whereas plasma concentrations of ARA or DHA alone were not [38]. Heude’s four-year cohort study involving 246 elderly people indicated that elderly subjects with high *n*-6 PUFA and low *n*-3 PUFA in their erythrocytes were most likely to experience cognitive decline [39]. Therefore, the ratio of dietary *n*-3/*n*-6 PUFA intake may influence the potential role of PUFAs on cognitive decline and dementia, the optimal ratio of *n*-6/*n*-3 for a healthier diet should be <5/1 [40].

In the present study, we observed a correlation between the erythrocyte membrane DHA proportion with the total MoCA score (*r* = 0.356) in elderly adults with cognition decline. This result indicates that a reduction in erythrocyte membrane DHA content might predict the cognition decline in elderly adults. Despite the uncertain effects of dietary DHA supplement on the prevention of cognition decline in population-based clinical intervention, an increasing amount of evidence support the beneficial effects of a DHA-rich diet on cognition. In experimental animals, learning and cognitive behavior are related to brain DHA status, which, in turn, is related to the levels of dietary *n*-3 PUFA [41]. In other transgenic AD mouse models, DHA also protects against dendritic pathology; the reduction of dietary *n*-3 PUFA in an AD mouse model resulted in 80%–90% losses of the postsynaptic actin-regulating protein drebrin in the brain [42]. Nonetheless, up to date, no definitive dietary recommendations on DHA consumption in relation to the risk for dementia and cognitive decline are possible. Since the content of in vivo DHA were determined largely by dietary *n*-3 PUFA intake, we suggest that the consumption of a DHA-rich diet should be encouraged to reduce the risk of cognitive impairment and, subsequently, disability in the elderly.

Several limitations to the present study should be considered. The small sampling number is a drawback of the present study. Moreover, the present study is a cross-sectional study, and some potential uncontrolled covariates were possible during data analysis. Therefore, a large-scale cohort study is required to clarify the relationship between the in vivo fatty acid profile and cognition in the elderly. Additionally, the participants of the current study were mainly elderly Chinese adults. The variable of dietary pattern on the erythrocyte fatty acid profile should not be neglected. Therefore, our results are not necessarily generalizable to other populations.

## 5. Conclusions 

In conclusion, the data of the present study indicated that MCI subjects have lower erythrocyte unsaturated fatty acids than control subjects. Lower erythrocyte unsaturated fatty acid (20:4 *n*-6, 20:5 *n*-3, DHA, total *n*-3 PUFA) and higher saturated fatty acid (12:0, 18:0, 22:0, total SFA) proportions might predict the decline of cognitive function in elderly Chinese adults.

## Figures and Tables

**Table 1:** Demographics, lifestyle characteristics, and clinical parameters of the participants.

	Control (*n* = 60)	MCI (*n* = 60)	*p* Value
Demographic Characteristics			
Age (year), mean ± SD	64.33 ± 6.0	64.15 ± 6.35	0.90
Gender, male (%)	42.5	42.5	0.59
Education, n (%)			0.036
Illiterate	3 (5)	8 (13.3)	
Primary school	16 (26.7)	21 (35)	
Junior high school	27 (45)	17 (28.3)	
High school	9 (15)	10 (16.7)	
Junior college	1 (1.7)	2 (3.3)	
Undergraduate and above	4 (6.7)	2 (3.3)	
Lifestyle (%)			
Smoking (%)	10 (17.5)	12 (20)	0.97
Alcohol Drinking (%)	15 (25)	18 (30)	0.40
**Clinical Parameters (mean ± SD)**			
BMI (kg/m^2^)	26.07 ± 2.91	25.09 ± 3.07	0.15
Glu (mmol/L)	5.37 ± 1.29	5.43 ± 3.07	0.85
TG (mmol/L)	1.88 ± 1.68	1.61 ± 0.90	0.38
TC (mmol/L)	4.85 ± 1.09	4.71 ± 1.14	0.59
HDL-C (mmol/L)	1.29 ± 0.45	1.26 ± 0.33	0.73
LDL-C (mmol/L)	3.27 ± 0.92	3.09 ± 0.99	0.41

BMI: body mass index; MCI: mild cognitive impairment; Glu: glucose; TG: triglyceride; TC: total cholesterol; LDL-C: low density lipoprotein cholesterol; HDL-C: high density lipoprotein cholesterol. The chi-square test was applied for the analysis of binary categorical variables between normal and MCI groups. A Student’s *t*-test was used for the data analysis of clinical biomarkers. *p* < 0.05 was considered to be statistically significant.

**Table 2:** Intake of lipid-rich foods of MCI patients and control subjects (g/day).

Foods Item	Control (*n* = 60)	MCI (*n* = 60)	*p* Value
Red meat	49.74 ± 6.47	63.75 ± 6.29	0.385
Poultry	16.09 ± 2.78	26.61 ± 2.71	0.008
Fish	50.10 ± 4.68	35.48 ± 4.54	0.027
Egg	36.71 ± 2.38	39.70 ± 2.31	0.864
Milk	229.43 ± 12.49	215.58 ± 11.87	0.424
Nuts	26.37 ± 4.08	25.46 ± 3.29	0.862
Legume	62.17 ± 6.98	50.83 ± 6.78	0.175
Cooking oil	35.29 ± 4.01	35.10 ± 3.90	0.826

Data were expressed as mean ± SD; Student *t*-test method was used for data analysis. *p <* 0.05 was considered to be statistically significant.

**Table 3:** Erythrocyte fatty acid profile of control and MCI subjects (*w*/*w*, %).

Fatty Acid	Control (*n* = 60)	MCI (*n* = 60)	*p* Value
12:0	0.44 ± 0.02	0.40 ± 0.03	0.262
14:0	0.60 ± 0.02	0.59 ± 0.02	0.666
15:0	0.23 ± 0.01	0.21 ± 0.01	0.134
16:0	29.69 ± 0.46	29.99 ± 0.57	0.681
18:0	14.92 ± 0.22	15.14 ± 0.25	0.499
20:0	0.43 ± 0.01	0.42 ± 0.01	0.716
22:0	1.79 ± 0.06	1.80 ± 0.07	0.968
23:0	0.26 ± 0.01	0.26 ± 0.01	0.920
24:0	4.74 ± 0.16	4.80 ± 0.20	0.804
Total SFA	53.52 ± 0.86	53.70 ± 1.00	0.894
16:1 *n*-7	0.21 ± 0.02	0.24 ± 0.03	0.165
18:1 *n*-9	15.80 ± 0.41	16.04 ± 0.41	0.685
22:1 *n*-9	1.00 ± 0.13	0.73 ± 0.14	0.164
24:1 *n*-9	3.19 ± 0.11	3.20 ± 0.12	0.952
Total MUFAs	19.96 ± 0.42	20.42 ± 0.42	0.449
18:2 *n*-6	11.19 ± 0.30	11.85 ± 0.62	0.310
20:3 *n*-6	1.11 ± 0.05	1.18 ± 0.11	0.525
20:4 *n*-6	12.26 ± 0.56	10.64 ± 0.55	0.047
Total *n*-6 PUFAs	24.07 ± 0.88	22.78 ± 0.83	0.308
20:5 *n*-3	0.26 ± 0.02	0.21 ± 0.01	0.022
22:6 *n*-3	3.01 ± 0.14	2.57 ± 0.19	0.056
Total *n*-3 PUFAs	3.27 ± 0.15	2.78 ± 0.20	0.043
Total PUFAs	27.21 ± 1.01	26.27 ± 1.07	0.529
*n*-6/*n*-3 ratio	7.94 ± 0.28	8.86 ± 0.51	0.094

Data were expressed as mean ± SE. Student *t*-test method was applied for comparing the differences of erythrocyte fatty acids in control and MCI subjects. SFA: saturated fatty acid; PUFAs: polyunsaturated fatty acids. *p <* 0.05 was considered to be statistically significant.

**Table 4:** Correlation coefficient (*r*) between erythrocyte fatty acids and cognitive function in MCI subjects (*n* = 60).

Cognition	12:0	18:0	22:0	Total SFA	18:2 *n*-6	24:4 *n*-6	Total *n*-6	20:5 *n*-3	22:6 *n*-3	Total PUFAs	*n*-6/*n*-3 Ratio
Visual-spatial ability	–0.096	–0.259	–0.323 *	–0.311 *	0.302 *	0.194	0.341 *	0.080	0.091	0.173	–0.096
Name	–0.140	–0.287	–0.194	–0.280	0.305 *	0.130	0.240	–0.032	0.001	0.174	–0.053
Attention	0.097	0.105	–0.010	0.028	–0.248	0.022	–0.097	0.206	0.135	–0.103	–0.006
Language	0.149	–0.080	–0.049	–0.051	–0.059	0.010	0.049	0.112	–0.038	0.005	0.039
Abstraction	–0.069	0.096	–0.025	–0.165	0.100	–0.122	0.102	0.107	–0.005	0.211	–0.076
Delayed memory	0.025	–0.334 *	0.117	–0.046	0.145	–0.245	–0.179	–0.098	–0.285	0.236	0.274
Orientation	0.022	–0.369 *	–0.168	–0.141	0.265	0.025	0.118	–0.032	–0.008	0.235	–0.056
Total score	–0.450 *	–0.008	–0.415 *	–0.322	0.091	0.434 *	0.286	–0.091	0.356 *	–0.219	–0.199

SFA: saturated fatty acid; PUFAs: polyunsaturated fatty acids. * *p <* 0.05.

## References

[1] Coley N., Vaurs C., Andrieu S. (2015). Nutrition and cognition in aging adults. Clin. Geriatr. Med..

[2] Solfrizzi V., Frisardi V., Capurso C., D’Introno A., Colacicco A.M., Vendemiale G., Capurso A., Panza F. (2010). Dietary fatty acids in dementia and predementia syndromes: Epidemiological evidence and possible underlying mechanisms. Ageing Res. Rev..

[3] Freund-Levi Y., Eriksdotter-Jönhagen M., Cederholm T., Basun H., Faxén-Irving G., Garlind A., Vedin I., Vessby B., Wahlund L.O., Palmblad J. (2006). Omega-3 fatty acid treatment in 174 patients with mild to moderate Alzheimer disease: OmegAD study: A randomized double-blind trial. Arch. Neurol..

[4] Solfrizzi V., Capurso C., D’Introno A., Colacicco A.M., Frisardi V., Santamato A., Ranieri M., Fiore P., Vendemiale G., Seripa D. (2008). Dietary fatty acids, age-related cognitive decline, and mild cognitive impairment. J. Nutr. Health Aging.

[5] Solfrizzi V., D’Introno A., Colacicco A.M., Capurso C., Del Parigi A., Capurso S., Gadaleta A., Capurso A., Panza F. (2005). Dietary fatty acids intake: Possible role in cognitive decline and dementia. Exp. Gerontol..

[6] Huang M.C., Brenna J.T., Chao A.C., Tschanz C., Diersen-Schade D.A., Hung H.C. (2007). Differential tissue dose responses of (*n*-3) and (*n*-6) PUFA in neonatal piglets fed docosahexaenoate and arachidonoate. J. Nutr..

[7] Conquer J.A., Tierney M.C., Zecevic J., Bettger W.J., Fisher R.H. (2000). Fatty acid analysis of blood plasma of patients with Alzheimer’s disease, other types of dementia and cognitive impairment. Lipids.

[8] Sinn N., Milte C.M., Street S.J., Buckley J.D., Coates A.M., Petkov J., Howe P.R. (2012). Effects of *n*-3 fatty acids, EPA v. DHA, on depressive symptoms, quality of life, memory and executive function in older adults with mild cognitive impairment: A 6-month randomised controlled trial. Br. J. Nutr..

[9] Chiu C.C., Su K.P., Cheng T.C., Liu H.C., Chang C.J., Dewey M.E., Stewart R., Huang S.Y. (2008). The effects of omega-3 fatty acids monotherapy in Alzheimer’s disease and mild cognitive impairment: A preliminary randomized double-blind placebo-controlled study. Prog. Neuropsychopharmacol. Biol. Psychiatry.

[10] Pottala J.V., Yaffe K., Robinson J.G., Espeland M.A., Wallace R., Harris W.S. (2014). Higher RBC EPA + DHA correspond with larger total brain and hippocampal volumes: WHIMS-MRI study. Neurology.

[11] Brenner S.R. (2012). Red blood cell omega-3 fatty acid levels and markers of accelerated brain aging. Neurology.

[12] Lu J., Li D., Li F., Zhou A., Wang F., Zuo X., Jia X.F., Song H., Jia J. (2011). Montreal cognitive assessment in detecting cognitive impairment in elderly Chinese individuals: A population-based study. J. Geriatr. Psychiatry Neurol..

[13] Zhang W., Li Q., Shi L., Lu K., Shang Q., Yao L., Ye G. (2009). Investigation of dietary intake of cadmium in certain polluted area of south in China. Wei Sheng Yan Jiu.

[14] Hata Y., Nakajima K. (1986). Application of friedewald’s LDL-cholesterol estimation formula to serum lipids in the Japanese population. Jpn. Circ. J..

[15] Folch J., Ascoli I., Lees M., Meath J.A., Lebaron N. (1951). Preparation of lipids extracts from brain tissue. J. Biol. Chem..

[16] Dai X.W., Zhang B., Wang P., Chen G.G., Chen Y.M., Su Y.X. (2014). Erythrocyte membrane *n*-3 fatty acid levels and carotid atherosclerosis in Chinese men and women. Atherosclerosis.

[17] Zhang B., Wang P., Chen C.G., He Q.Q., Zhuo S.Y., Chen Y.M., Su Y.X. (2010). Validation of an FFQ to estimate the intake of fatty acids using erythrocyte membrane fatty acids and multiple 3d dietary records. Public Health Nutr..

[18] Lim S.L., Gao Q., Nyunt M.S., Gong L., Lunaria J.B., Lim M.L., Ling A., Lam C.S., Richards A.M., Ling L.H. (2015). Vascular health indices and cognitive domain function: Singapore longitudinal ageing studies. J. Alzheimers Dis..

[19] Kim T.W., Song I.U., Jeong D.S., Lee K.S. (2016). Clinical effect of cerebrovascular atherosclerosis on cognition in Alzheimer’s disease. Arch Gerontol. Geriatr..

[20] Bajerska J., Woźniewicz M., Suwalska A., Jeszka J. (2014). Eating patterns are associated with cognitive function in the elderly at risk of metabolic syndrome from rural areas. Eur. Rev. Med. Pharmacol. Sci..

[21] Van de Rest O., Berendsen A.A., Haveman-Nies A., de Groot L.C. (2015). Dietary patterns, cognitive decline, and dementia: A systematic review. Adv. Nutr..

[22] Morris M.C., Evans D.A., Bienias J.L., Tangney C.C., Bennett D.A., Wilson R.S., Aggarwal N., Schneider J. (2003). Consumption of fish and *n*-3 fatty acids and risk of incident Alzheimer disease. Arch. Neurol..

[23] Morris M.C., Evans D.A., Tangney C.C., Bienias J.L., Wilson R.S. (2005). Fish consumption and cognitive decline with age in a large community study. Arch. Neurol..

[24] Solfrizzi V., Frisardi V., Seripa D., Capurso C., Vendemiale G., Pilotto A., Panza F. (2010). Dietary patterns and protection against Alzheimer disease and cognitive decline. Arch. Neurol..

[25] Danthiir V., Hosking D., Burns N.R., Wilson C., Nettelbeck T., Calvaresi E., Clifton P., Wittert G.A. (2014). Cognitive performance in older adults is inversely associated with fish consumption but not erythrocyte membrane *n*-3 fatty acids. J. Nutr..

[26] Soni M., Rahardjo T.B., Soekardi R., Sulistyowati Y., Lestariningsih, Yesufu-Udechuku A., Irsan A., Hogervorst E. (2014). Phytoestrogens and cognitive function: A review. Maturitas.

[27] Franco O.H., Burger H., Lebrun C.E., Peeters P.H., Lamberts S.W., Grobbee D.E., van der Schouw Y.T. (2005). Higher dietary intake of lignans is associated with better cognitive performance in postmenopausal women. J. Nutr..

[28] Milte C.M., Sinn N., Street S.J., Buckley J.D., Coates A.M., Howe P.R. (2011). Erythrocytepolyunsaturated fatty acid status, memory, cognition and mood in older adultswith mild cognitive impairment and healthy controls. Prostaglandins Leukot. Essent. Fatty Acids.

[29] Upadhyay R.K. (2015). Emerging risk biomarkers in cardiovascular diseases anddisorders. J. Lipids.

[30] Cunnane S.C., Schneider J.A., Tangney C., Tremblay-Mercier J., Fortier M., Bennett D.A., Morris M.C. (2012). Plasma and brain fatty acid profiles in mild cognitive impairment and Alzheimer’s disease. J. Alzheimers Dis..

[31] Iuliano L., Pacelli A., Ciacciarelli M., Zerbinati C., Fagioli S., Piras F., Orfei M.D., Bossù P., Pazzelli F., Serviddio G. (2013). Plasma fatty acid lipidomics in amnestic mild cognitive impairment and Alzheimer’s disease. J. Alzheimers Dis..

[32] Morris M.C., Evans D.A., Bienias J.L., Tangney C.C., Bennett D.A., Aggarwal N., Schneider J., Wilson R.S. (2003). Dietary fats and the risk of incident Alzheimerdisease. Arch. Neurol..

[33] Laitinen M.H., Ngandu T., Rovio S., Helkala E.-L., Uusitalo U., Viitanen M., Nissinen A., Tuomilehto J., Soininen H., Kivipelto M. (2006). Fat intake at midlife and risk of dementia and Alzheimer’s disease: Apopulation-based study. Dement. Geriatr. Cogn. Disord..

[34] Kalmijn S., Launer L.J., Ott A., Witteman J.C., Hofman A., Breteler M.M. (1997). Dietary fat intake and the risk of incident dementia in the Rotterdam study. Ann. Neurol..

[35] Baym C.L., Khan N.A., Monti J.M., Raine L.B., Drollette E.S., Moore R.D., Scudder M.R., Kramer A.F., Hillman C.H., Cohen N.J. (2014). Dietary lipids are differentially associated with hippocampal-dependent relational memory in prepubescent children. Am. J. Clin. Nutr..

[36] CorrêaLeite M.L., Nicolosi A., Cristina S., Hauser W.A., Nappi G. (2001). Nutrition and cognitive deficit in the elderly: A population study. Eur. J. Clin. Nutr..

[37] González S., Huerta J.M., Fernández S., Patterson A.M., Lasheras C. (2010). The relationshipbetween dietary lipids and cognitive performance in an elderly population. Int. J. Food Sci. Nutr..

[38] Samieri C., Feart C., Letenneur L., Dartigues J.F., Peres K., Auriacombe S., Peuchant E., Delcourt C., Barberger-Gateau P. (2008). Low plasma eicosapentaenoic acid and depressive symptomatology are independent predictors of dementia risk. Am. J. Clin. Nutr..

[39] Heude B., Ducimetière P., Berr C. (2003). EVA study. Cognitive decline and fatty acid composition of erythrocyte membranes—The EVA Study. Am. J. Clin. Nutr..

[40] De Lorgeril M., Salen P., Martin J.L., Monjaud I., Boucher P., Mamelle N. (1998). Mediterranean dietary pattern in a randomized trial: Prolonged survival and possible reduced cancer rate. Arch. Intern. Med..

[41] Moriguchi T., Greiner R.S., Salem N. (2000). Behavioral deficits associated with dietary induction of decreased brain docosahexaenoic acid concentration. J. Neurochem..

[42] Calon F., Lim G.P., Yang F., Morihara T., Teter B., Ubeda O., Rostaing P., Triller A., Salem N., Ashe K.H. (2004). Docosahexaenoic acid protects from dendritic pathology in an Alzheimer’s disease mouse model. Neuron.

